# Detection of TERT Promoter Mutations in Papillary Thyroid Carcinoma Using Droplet Digital PCR and Their Association with Aggressive Tumor Features

**DOI:** 10.3390/ijms27031497

**Published:** 2026-02-03

**Authors:** Jeongmin Lee, Chaiho Jeong, Jeonghoon Ha, Dong-Jun Lim, Tae-Jung Kim, Ki-Hyun Baek

**Affiliations:** 1 Division of Endocrinology and Metabolism, Department of Internal Medicine, Eunpyeong St. Mary’s Hospital, College of Medicine, The Catholic University of Korea, Seoul 03312, Republic of Korea; 082mdk45@catholic.ac.kr; 2 Division of Endocrinology and Metabolism, Department of Internal Medicine, Uijeongbu St. Mary’s Hospital, College of Medicine, The Catholic University of Korea, Uijeongbu 11765, Republic of Korea; cerbere@catholic.ac.kr; 3 Division of Endocrinology and Metabolism, Department of Internal Medicine, Seoul St. Mary’s Hospital, College of Medicine, The Catholic University of Korea, Seoul 06591, Republic of Korea; hajhoon@catholic.ac.kr (J.H.); ldj6026@catholic.ac.kr (D.-J.L.); 4 Department of Hospital Pathology, Yeouido St. Mary’s Hospital, College of Medicine, The Catholic University of Korea, Seoul 07345, Republic of Korea; 5 Division of Endocrinology and Metabolism, Department of Internal Medicine, Yeouido St. Mary’s Hospital, College of Medicine, The Catholic University of Korea, Seoul 07345, Republic of Korea

**Keywords:** thyroid neoplasm, TERT promoter mutation, polymerase chain reaction, thyroidectomy

## Abstract

This study evaluated the reliability of droplet digital polymerase chain reaction (ddPCR) for detecting TERT promoter (pTERT) mutations in formalin-fixed, paraffin-embedded (FFPE) thyroid cancer samples and examined their association with clinicopathological features. A retrospective cohort of 296 postoperative patients with papillary thyroid carcinoma (PTC) was analyzed. DNA extracted from archived FFPE thyroidectomy specimens was examined for TERT promoter mutations using ddPCR. pTERT mutations were detected in 14 cases (4.7%). Tumors harboring pTERT mutations were significantly larger than wild-type tumors (1.5 ± 1.3 cm vs. 1.0 ± 0.7 cm, *p* = 0.012) and showed higher frequencies of extrathyroidal extension (78.6% vs. 55.0%, *p* = 0.028), capsular invasion (85.7% vs. 63.1%, *p* = 0.036), and lymph node metastasis (64.3% vs. 44.0%, *p* = 0.012). Multivariate analysis demonstrated that increasing age (odds ratio (OR), 1.07; 95% confidence interval (CI), 1.01–1.13; *p* = 0.015), tumor size (OR, 1.86; 95% CI, 1.12–3.08; *p* = 0.016), and lymph node metastasis (OR, 3.50; 95% CI, 1.09–6.53; *p* = 0.026) were independently associated with pTERT mutations. ddPCR enables sensitive detection of pTERT mutations in archived FFPE thyroid cancer specimens and identifies tumors with aggressive clinicopathological features, supporting its utility for postoperative risk stratification in clinical practice.

## 1. Introduction

Papillary thyroid carcinoma (PTC) and follicular thyroid carcinoma are the predominant types of well-differentiated thyroid cancer (DTC), accounting for approximately 90% of all cases [1]. Despite the typically indolent nature of thyroid cancer and its favorable prognosis, lymph node (LN) metastases are observed in 20–31% of patients at the time of diagnosis. Furthermore, local recurrences occur in 5–20% of patients during post-treatment surveillance [2]. Although distant metastasis and poorly differentiated carcinoma are less common (6–23%), they are associated with a poor prognosis [3,4,5]. Notably, approximately one-third of patients with metastatic disease may experience disease stability for ≥10 years [6]. Given the long-term survival of most patients with DTC, identifying those who require aggressive treatment is crucial.

Clinicopathological parameters, including histopathological features, younger age, tumor size, LN invasion, vascular invasion, extrathyroidal extension, LN metastasis, and distant metastases, have been identified as predictive factors for thyroid cancer outcomes [7,8]. In addition, molecular testing for genetic alterations in thyroid cancer can enhance the diagnostic value of cytological examinations and improve the prediction of clinical outcomes. The integration of molecular and traditional methods offers the potential for improved patient stratification and individualized treatment. Several genetic alterations and mutations have been identified as key biomarkers for DTC, demonstrating varying sensitivities and specificities [9]. Despite significant attention given to the B-type raf proto-oncogene V600E (BRAF) mutation owing to its high prevalence in DTC [10], its association with recurrence and mortality remains inconsistent.

Mutations in the telomerase reverse transcriptase promoter (pTERT) have been identified in patients with DTC. TERT maintains telomere length and regulates cellular proliferation and immortality [11]. pTERT mutations are associated with increased malignancy, aggressive tumor behavior, treatment resistance, and poor clinical outcomes [12,13]. The two most commonly identified pTERT mutations (chr5:1295228C>T [C228T] and chr5:1295250C>T [C250T]) are found in 4.5–25.5% of DTCs and in 21.4–51.7% of poorly differentiated thyroid carcinomas [14,15,16]. Recent evidence has highlighted a synergistic effect when pTERT mutations co-occur with BRAF V600E, significantly enhancing tumor aggressiveness and worsening prognosis. These findings support integrative molecular models for more accurate risk stratification [17,18,19].

The major mutations in DTC are commonly identified using Sanger sequencing, allele-specific amplification polymerase chain reaction (PCR), quantitative PCR, pyrosequencing, and next-generation sequencing. However, these methods are expensive, require substantial quantities of nucleic acids, and show limited sensitivity in samples with low mutant allele frequencies. Their performance often declines in formalin-fixed, paraffin-embedded (FFPE) tissues owing to DNA fragmentation and chemical modifications introduced during tissue preservation. These limitations underscore the urgent need for highly sensitive and robust molecular assays optimized for the analysis of fragmented DNA derived from FFPE samples, as frequently encountered in routine thyroid cancer diagnostics.

Droplet digital PCR (ddPCR) is an emerging next-generation PCR technique characterized by high precision and sensitivity, allowing absolute quantification of nucleic acid target sequences even in samples with low DNA input [20]. In ddPCR, DNA samples are partitioned into thousands of nanoliter-sized water-in-oil droplets, each independently analyzed for specific genetic variants. Fluorescent probes that bind to either wild-type or mutant alleles enable accurate signal discrimination. This technique is highly sensitive, capable of detecting mutant allele frequencies as low as 0.5% and identifying as few as two mutant-positive droplets [21]. Recent liquid biopsy studies have demonstrated the expanding clinical utility of ddPCR for detecting low-frequency mutations, including pTERT and BRAF mutations, in circulating tumor DNA [22,23]. Although pTERT mutation detection in thyroid cancer tissue using ddPCR has been less extensively studied, the broader application of this platform in liquid biopsy and its integration into molecular prognostic models highlight important opportunities for further investigation. Differentiated thyroid cancer is characterized by an indolent clinical course and prolonged survival; however, clinically relevant events such as recurrence or metastasis may occur many years or even decades after initial treatment [24,25]. Consequently, long-term follow-up is essential, and retrospective molecular studies frequently rely on archived formalin-fixed, paraffin-embedded (FFPE) specimens obtained at the time of primary surgery. However, comprehensive next-generation sequencing (NGS) analyses are often limited in long-stored FFPE tissues because of progressive DNA fragmentation, fixation- and storage-related sequence artefacts, and reduced library complexity, particularly in GC-rich genomic regions such as the TERT promoter (pTERT) [26,27,28].

In this context, droplet digital PCR (ddPCR) represents a highly attractive alternative molecular platform. ddPCR enables absolute quantification of low-frequency variants using short amplicons optimized for fragmented FFPE-derived DNA and demonstrates robustness in samples unsuitable for NGS-based profiling. The novelty of the present study lies in demonstrating that ddPCR can reliably detect pTERT mutations in long-term archived postoperative thyroid cancer FFPE specimens, thereby providing clinically meaningful molecular information for retrospective risk stratification in a disease that intrinsically requires extended surveillance.

## 2. Results

### 2.1. Baseline Patient Characteristics

The median patient age was 47.8 ± 11.0 years (range, 17–78 years), and the majority of patients were female (n=233, 78.7%). Patient characteristics are summarized in Table 1. The mean tumor size was 1.0 ± 0.7 cm (range, 0.1–4.7 cm). Papillary thyroid microcarcinoma (<1 cm) accounted for 58.1% (172/296) of all cases.

Among the 296 patients evaluated, most were diagnosed with classic papillary thyroid carcinoma (PTC) (n=279, 94.3%). The tall cell variant of PTC was identified in eight patients (2.7%), whereas the follicular variant was observed in nine patients (3.0%). The mean thyroid-stimulating hormone level during postoperative follow-up was 3.29 ± 8.43 mIU/L, and the mean thyroglobulin antigen level was 1.96 ± 6.20 ng/mL.

### 2.2. Comparison of ddPCR Results Between pTERT Mutated Group and Wild-Type Group

pTERT mutations were detected by droplet digital PCR (ddPCR) in 14 patients (4.7%), including 10 patients with the C228T mutation and 4 patients with the C250T mutation. A comparison of clinicodemographic characteristics between the pTERT-mutated and wild-type groups is summarized in Table 2.

Sex distribution did not differ significantly between the two groups (*p* = 0.563), with a predominance of female patients in both the pTERT-mutated group (71.4%) and the wild-type group (79.1%). Patients in the pTERT-mutated group tended to be older; however, this difference did not reach statistical significance (53.1 ± 12.7 years vs. 47.6 ± 10.9 years, *p* = 0.065). Tumor size was significantly larger in the pTERT-mutated group than in the wild-type group (1.5 ± 1.3 cm vs. 1.0 ± 0.7 cm, *p* = 0.012).

In contrast, several pathological features differed significantly between the two groups. Extrathyroidal extension (78.6% vs. 55.0%, *p* = 0.038), capsular invasion (85.7% vs. 63.1%, *p* = 0.046), and lymph node metastasis (64.3% vs. 44.0%, *p* = 0.031) were significantly more frequent in the pTERT-mutated group. There was no significant difference in tumor multiplicity between the groups (42.9% vs. 28.7%, *p* = 0.059).

### 2.3. Clinicopathologic Parameters Associated with pTERT Mutations

Age was significantly associated with an increased risk of pTERT mutation (odds ratio (OR), 1.07; 95% confidence interval (CI), 1.01–1.13; *p* = 0.015), whereas female sex was not significantly associated with mutation status (OR, 1.08; 95% CI, 0.79–3.17; *p* = 0.731) (Table 3). Tumor size was also significantly associated with the presence of pTERT mutation (OR, 1.86; 95% CI, 1.12–3.08; *p* = 0.016).

Although relatively high odds ratios were observed for the associations of extrathyroidal extension, capsular invasion, and tumor multiplicity with pTERT mutations (OR range, 1.68–3.00), these associations did not reach statistical significance. In contrast, lymph node metastasis was significantly associated with pTERT mutation detected by droplet digital PCR (OR, 3.50; 95% CI, 1.71–6.53; *p* = 0.026).

## 3. Discussion

This study demonstrates that droplet digital PCR (ddPCR) is a reliable molecular approach for detecting pTERT mutations in formalin-fixed, paraffin-embedded (FFPE) thyroid cancer samples and that these mutations are associated with aggressive clinicopathological features, including larger tumor size and lymph node (LN) metastasis, in differentiated thyroid cancer (DTC). Using ddPCR, pTERT mutations were identified in 4.7% of papillary thyroid carcinoma (PTC) cases in this cohort. Previous studies have reported mutation frequencies ranging from 0% to 8.7% in papillary thyroid microcarcinomas [29,30,31]. Given that papillary thyroid microcarcinomas accounted for 58.1% of cases in the present cohort, the observed mutation frequency is consistent with existing literature [29,30,31]. Variability across studies likely reflects differences in case composition and disease aggressiveness, as pTERT mutations are more prevalent in advanced-stage and biologically aggressive tumors.

The high proportion of papillary thyroid microcarcinomas in this cohort warrants careful interpretation of group comparisons. Because microcarcinomas are generally associated with indolent clinical behavior, the mutation-negative group may be enriched for small, low-risk tumors, potentially attenuating apparent differences in aggressiveness between groups. This cohort composition may therefore influence the magnitude of observed associations and should be considered when interpreting clinicopathological correlations [18,19,32].

In the present study, extrathyroidal extension and capsular invasion were significantly more frequent in the pTERT mutation-positive group in univariate analyses but did not remain significant after adjustment in multivariate models. This discrepancy likely reflects the strong interrelationship among aggressive clinicopathological features in papillary thyroid carcinoma, including tumor size, lymph node metastasis, and local invasive characteristics. When correlated variables are simultaneously included in multivariate analyses, the apparent independent contribution of individual pathological features may be attenuated.

Residual confounding by unmeasured molecular factors cannot be excluded. In particular, because BRAF V600E status was not assessed in this cohort, potential interactions or confounding effects related to co-mutation status may have influenced multivariate estimates. Notably, pTERT mutations remained independently associated with lymph node metastasis, suggesting a closer relationship with metastatic potential than with local invasive features after adjustment for correlated clinicopathological variables.

However, the absence of BRAF V600E data precludes evaluation of the well-established synergistic effects between pTERT and BRAF mutations, which have been shown to markedly worsen prognosis in thyroid cancer. This limitation fundamentally restricts interpretation of the prognostic power of pTERT mutations alone. Accordingly, the clinical implications of pTERT mutations should be interpreted within the broader molecular landscape of high-risk thyroid neoplasms, and future studies incorporating integrated co-mutation profiling will be required to determine whether the observed associations remain independent after accounting for BRAF status [19,32].

In addition, the relatively small number of pTERT mutation-positive cases limited statistical power and resulted in wide confidence intervals, which may have further reduced the ability to detect independent associations in multivariate analyses. Similar observations have been reported in previous thyroid cancer studies, in which aggressive pathological features showed significance in univariate analyses but lost statistical significance after multivariable adjustment [33].

Consistent with prior reports, the C228T mutation was detected more frequently than the C250T mutation, and these two hotspot mutations did not co-occur in the same patient [14,34,35]. This distribution supports the dominant role of C228T as the primary pTERT alteration in thyroid cancer and underscores the biological relevance of targeting these hotspot mutations in molecular diagnostics. Although clinicopathological associations were explored by mutation subtype, meaningful subtype-specific comparisons were not feasible because the number of C250T-positive cases was small (*n* = 4). Larger cohorts will be required to determine whether C228T and C250T confer differential clinicopathological impacts.

The association between pTERT mutations and aggressive tumor behavior is biologically plausible given the fundamental role of telomerase reverse transcriptase in telomere maintenance, cellular immortality, and proliferative capacity [36]. In thyroid cancer, telomerase activation facilitates evasion of replicative senescence and apoptosis, thereby promoting sustained oncogenic transformation [37]. Increased telomerase activity resulting from pTERT mutations may contribute to invasive growth patterns and increased metastatic potential [38]. Nevertheless, molecular analysis of the TERT promoter region remains technically challenging because of its high GC content, which can interfere with amplification and sequencing efficiency [39].

In this context, ddPCR represents a robust molecular platform for pTERT mutation analysis, particularly in archived FFPE specimens. By partitioning DNA molecules into thousands of independent reaction compartments, ddPCR enables absolute quantification and reliable detection of target sequences from fragmented DNA [40,41]. Importantly, however, direct head-to-head comparative studies evaluating analytical sensitivity between ddPCR and conventional PCR-based assays specifically for pTERT mutations in thyroid cancer are limited. Therefore, ddPCR should be regarded as one of several viable molecular approaches rather than a universally superior method.

This consideration is particularly relevant in differentiated thyroid cancer, in which clinically meaningful endpoints such as recurrence or metastasis may emerge only after prolonged follow-up [24,25]. As a result, archived FFPE tissues often represent the only available material for correlating molecular alterations with long-term clinical outcomes. While next-generation sequencing (NGS) offers comprehensive genomic profiling, its performance may be compromised in older FFPE blocks because of DNA degradation, fixation-induced artefacts, and reduced library complexity [26,27,28]. In such settings, ddPCR provides a pragmatic and complementary strategy for targeted molecular analysis when sequencing-based approaches are technically constrained [42,43].

From a clinical perspective, pTERT mutation testing may provide added value for postoperative risk stratification, analogous to the clinical use of BRAF V600E testing in thyroid cancer. Although ddPCR offers certain technical advantages, pTERT mutation testing does not necessarily require ddPCR in all clinical settings. Conventional PCR-based methods, Sanger sequencing or SNaPshot assays may also be applied as reflex tests in routine diagnostic practice when tumor content and assay performance are appropriate [44]. From a cost–benefit standpoint, assay selection should balance analytical performance, turnaround time, infrastructure requirements, and the clinical question being addressed. A tiered or reflex testing strategy may therefore represent a pragmatic approach to integrating pTERT testing into routine care [32].

Several limitations of this study should be acknowledged. First, the retrospective design precludes causal inference, and prospective studies are required to confirm the prognostic value of pTERT mutations detected by ddPCR. Second, the single-center nature of the cohort and inclusion of only surgically treated patients may limit generalizability. Third, although cutoff values for ddPCR mutation detection were adapted from established glioma protocols, tumor-specific optimization of these thresholds may further improve diagnostic accuracy [39,45]. Because analytical decision thresholds in digital PCR should be established for the intended tumor type and specimen matrix [46,47], thyroid-specific cutoff validation remains necessary. Given the limited number of mutation-positive cases, such validation is deferred to future larger-scale studies.

Finally, the absence of longitudinal follow-up data, including recurrence-free survival or disease-specific mortality, precluded direct confirmation of the long-term clinical utility of ddPCR-based pTERT testing for outcome prediction. Nonetheless, given the consistent association between pTERT mutations and adverse outcomes across multiple tumor types [15,34,48,49,50,51], the observed correlations with aggressive pathological features support the biological and clinical relevance of pTERT mutations in thyroid cancer. Future multicenter studies incorporating long-term follow-up and integrated molecular profiling are warranted to further define their prognostic utility.

## 4. Materials and Methods

### 4.1. Study Design, Patients, and Sample Collection

**Figure 1:**
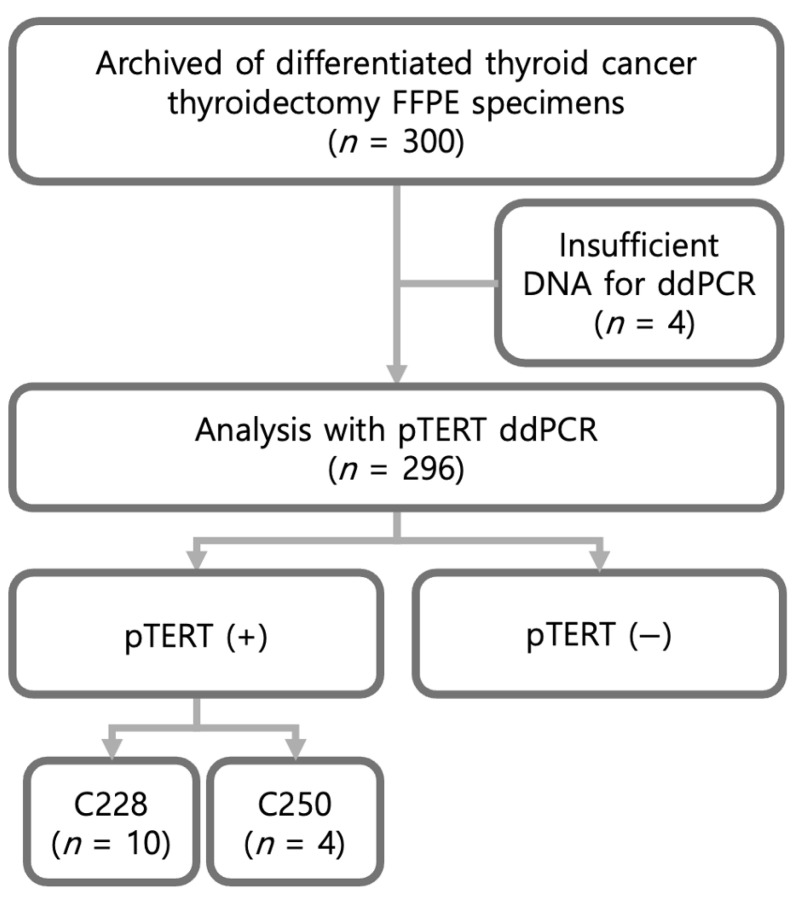
Flowchart of patient inclusion and ddPCR analysis.

This retrospective study included archived formalin-fixed, paraffin-embedded (FFPE) thyroid cancer specimens and corresponding clinicopathological data from 300 patients with differentiated thyroid cancer (DTC) who had undergone thyroidectomy between March 2010 and July 2012. Samples with insufficient DNA for droplet digital PCR (ddPCR) analysis (n=4) were excluded; therefore, 296 cases were ultimately evaluated (Figure 1). Among these 296 cases, ddPCR identified 14 mutation-positive samples (C228T, n=10; C250T, n=4) and 282 mutation-negative samples.

### 4.2. Digital Droplet PCR

#### 4.2.1. DNA Extraction

Genomic DNA was extracted from formalin-fixed, paraffin-embedded (FFPE) tissue sections using the MoBio FFPE DNA Isolation Kit (Qiagen, Hilden, Germany) according to the manufacturer’s instructions. Briefly, 5–10 μm tissue sections were carefully cut and deparaffinized using xylene followed by graded ethanol washes. Protein digestion was performed with proteinase K at 56 °C until complete tissue lysis was achieved. DNA was subsequently purified and eluted in nuclease-free water. DNA concentration and purity were assessed using a NanoDrop 2000C spectrophotometer (Thermo Fisher Scientific, Waltham, MA, USA). DNA integrity was evaluated using the DNA Integrity Number (DIN) measured by the Agilent TapeStation, with a DIN value of ≥3 considered acceptable for high-quality droplet digital PCR (ddPCR) analysis [52].

#### 4.2.2. pTERT Mutation Validation

pTERT mutations were validated by PCR amplification of the pTERT region using HotStarTaq Plus DNA Polymerase (Qiagen, Hilden, Germany). The primer sequences were as follows: forward, 5′-CAGGGAGCAATGCGTCCTCGGGTTC-3′; reverse, 5′-GCGCTGCCTGAAACTCGC-3′. PCR amplification was performed under the following cycling conditions: initial denaturation at 95 °C for 5 min; 40 cycles of denaturation at 95 °C for 45 s, annealing at 65 °C for 45 s, and extension at 72 °C for 1 min; followed by a final extension at 72 °C for 10 min and a hold at 4 °C. PCR products were purified and sequenced by Genewiz (South Plainfield, NJ, USA) or analyzed using SNaPshot assays with custom primers and reagents from Thermo Fisher Scientific (Waltham, MA, USA).

#### 4.2.3. ddPCR Primer and Probe Design

Mutation-specific assays targeting the hotspot pTERT mutations C228T and C250T, along with corresponding wild-type assays, were obtained from Bio-Rad Laboratories (Pleasanton, CA, USA). These assays generated short amplicons ranging from 88 to 113 bp, optimized for fragmented DNA derived from FFPE tissues. Mutation-specific hydrolysis probes were labeled with FAM, whereas wild-type probes were labeled with HEX, enabling duplex detection. The assays were validated to ensure high specificity and minimal cross-reactivity between mutant and wild-type alleles under optimized ddPCR conditions.

#### 4.2.4. ddPCR Reaction Mix and Droplet Generation

Each ddPCR reaction was prepared in a final volume of 20 μL, containing 10 μL of ddPCR Supermix for Probes (No dUTP; Bio-Rad), 1 μL of 20× TERT primer/probe assay, 1 μL of CviQI restriction enzyme, 10–20 ng of FFPE-derived genomic DNA, and nuclease-free water to reach the final volume. To enhance amplification efficiency in the GC-rich pTERT region, EDTA (final concentration, 1 mM) and betaine (0.75–1.0 M) were added to the reaction mixture.

Droplets were generated using the QX200 Droplet Generator (Bio-Rad) according to the manufacturer’s instructions. The resulting droplet emulsions were transferred to a 96-well PCR plate and sealed with pierceable foil prior to thermal cycling.

#### 4.2.5. ddPCR Thermal Cycling

PCR amplification was performed on a C1000 Touch Thermal Cycler (Bio-Rad) using the following optimized cycling conditions: initial denaturation at 95 °C for 10 min; 50 cycles of denaturation at 96 °C for 30 s and annealing/extension at 60 °C for 1 min; followed by enzyme deactivation at 98 °C for 10 min and a hold at 4 °C. These conditions were determined based on optimization experiments, including annealing temperature gradients, DNA input titration, and betaine concentration adjustments.

#### 4.2.6. Droplet Reading and Data Analysis

After amplification, droplets were analyzed using the QX200 Droplet Reader (Bio-Rad). Fluorescence data were processed with QuantaSoft software (version 1.7; Bio-Rad). Positive and negative droplets were discriminated by manually setting fluorescence thresholds based on no-template controls, wild-type controls, and positive mutant controls.

#### 4.2.7. Quality Control and Mutation Calling

Each ddPCR plate included a no-template control (NTC) and wild-type (WT) control in every run (i.e., per sample plate rather than intermittently). These controls were used to set fluorescence thresholds and to monitor background signal and potential contamination for each run. Quality control criteria required each sample to generate at least 12,000 accepted droplets. A sample was classified as mutation-positive when the number of FAM- or HEX-positive droplets exceeded the upper limit of background signals observed in wild-type controls. All experiments were conducted using a unidirectional workflow, aerosol-resistant pipette tips, and physical separation of pre- and post-PCR work areas to minimize contamination [42].

#### 4.2.8. Cutoff Values for ddPCR

Given the pathological and molecular similarities between papillary thyroid carcinoma and glioma, cutoff values for ddPCR mutation detection were adapted from established glioma protocols. Specifically, the lower limits of reliable quantification for the C228T and C250T mutations were set at ≥1.0% and ≥1.5% mutant allele frequency, respectively. These thresholds were selected based on prior studies demonstrating their effectiveness in minimizing false-positive results while maintaining sensitivity for detecting low-frequency mutations in GC-rich promoter regions [45]. The selected cutoff values provided an optimal balance between analytical sensitivity and specificity for clinically relevant mutation detection.

### 4.3. Statistical Analysis

Continuous variables are presented as the mean ± standard deviation (SD), whereas categorical variables are expressed as numbers and percentages. Associations between clinicopathological variables and droplet digital PCR (ddPCR) positivity were assessed using univariate and multivariate logistic regression analyses.

Univariate logistic regression was performed to evaluate the association between individual clinicopathological variables (age, sex, tumor size, extrathyroidal extension, capsular invasion, tumor multiplicity, and lymph node metastasis) and pTERT mutation status. Multivariate logistic regression was then conducted to identify independent factors associated with pTERT mutation status, with variables selected a priori based on clinical relevance and potential confounding.

All statistical analyses were performed using SAS software version 9.4 (SAS Institute Inc., Cary, NC, USA). A two-sided *p*-value of <0.05 was considered statistically significant.

## 5. Conclusions

In this retrospective cohort of archived FFPE papillary thyroid carcinoma specimens, ddPCR enabled sensitive detection of pTERT mutations and identified a subset of tumors associated with aggressive clinicopathological features. Notably, pTERT mutations were independently associated with lymph node metastasis, supporting the potential utility of ddPCR-based pTERT testing for postoperative risk stratification using archival tissue. Future multicenter studies incorporating long-term outcomes and integrated co-mutation profiling are warranted to further define the prognostic value of pTERT mutations in thyroid cancer.

## Figures and Tables

**Table 1:** Baseline patient characteristics (n=296).

Clinical Parameters	Value
Age, years	47.8 ± 11.0 (range, 17–78)
Gender	
Male, *n* (%)	63 (21.3)
Female, *n* (%)	233 (78.7)
Tumor size, cm (mean ± SD)	1.0 ± 0.7 (range, 0.1–4.7)
Papillary thyroid microcarcinoma, *n* (%)	172 (58.1)
Lymph node metastasis, *n* (%)	138 (46.6)
Histology	
Classic PTC, *n* (%)	279 (94.3)
Tall cell variant, *n* (%)	8 (2.7)
Follicular thyroid carcinoma, *n* (%)	9 (3.0)
TSH, mIU/L (mean ± SD)	3.29 ± 8.43
Thyroglobulin, ng/mL (mean ± SD)	1.96 ± 6.20

Abbreviations: TSH, thyroid-stimulating hormone; PTC, papillary thyroid carcinoma.

**Table 2:** Comparison of clinicopathological characteristics according to pTERT mutation status.

Clinical Parameters	pTERT (+) (*n* = 14)	pTERT (–) (*n* = 282)	*p*-Value
Female/Male, *n* (%)	10 (71.4)/4 (28.6)	223 (79.1)/59 (20.9)	0.563
Age, years	53.1 ± 12.7	47.6 ± 10.9	0.065
Tumor size, cm	1.5 ± 1.3	1.0 ± 0.7	0.012
C228T mutation, *n* (%)	10 (71.4)	0	NA
C250T mutation, *n* (%)	4 (28.6)	0	NA
Extrathyroidal extension, *n* (%)	11 (78.6)	155 (55.0)	0.038
Capsular invasion, *n* (%)	12 (85.7)	178 (63.1)	0.046
Multiplicity, *n* (%)	6 (42.9)	81 (28.7)	0.059
Lymph node metastasis, *n* (%)	9 (64.3)	124 (44.0)	0.031

Abbreviations: pTERT, telomerase reverse transcriptase promoter; NA, not applicable.

**Table 3:** Multivariate logistic regression analysis of variables associated with pTERT mutations.

Parameters	OR (95% CI)	*p*-Value
Age (per year)	1.07 (1.01–1.13)	0.015
Female sex	1.08 (0.79–3.17)	0.731
Tumor size	1.86 (1.12–3.08)	0.016
Extrathyroidal extension	3.00 (0.82–10.99)	0.418
Capsular invasion	2.21 (0.17–2.45)	0.244
Tumor multiplicity	1.68 (0.49–5.52)	0.418
Lymph node metastasis	3.50 (1.71–6.53)	0.026

OR, odds ratio; CI, confidence interval.

## Data Availability

Data available upon request due to restrictions.

## Abbreviations

The following abbreviations are used in this manuscript:

PTC	Papillary thyroid carcinoma
DTC	Differentiated thyroid cancer
LN	Lymph node
BRAF	B-type raf proto-oncogene V600E
pTERT	TERT promoter
PCR	Polymerase chain reaction
ddPCR	Droplet digital PCR
FFPE	Formalin-fixed paraffin-embedded
IRB	Institutional Review Board
OR	odds ratios
CI	confidence intervals
